# Rehabilitation strategies for optimisation of functional recovery after major joint replacement

**DOI:** 10.1186/s40634-018-0156-2

**Published:** 2018-10-11

**Authors:** Thomas Bandholm, Thomas W. Wainwright, Henrik Kehlet

**Affiliations:** 1 Physical Medicine & Rehabilitation Research – Copenhagen (PMR-C), Department of Occupational and Physical Therapy, Amager-Hvidovre Hospital, University of Copenhagen, Kettegaards Alle 30, DK-2650 Hvidovre, Denmark; 2 Department of Orthopedic Surgery, Amager-Hvidovre Hospital, University of Copenhagen, Kettegaards Alle 30, DK-2650 Hvidovre, Denmark; 3 Clinical Research Centre (056), Amager-Hvidovre Hospital, University of Copenhagen, Kettegaards Alle 30, DK-2650 Hvidovre, Denmark; 4 Orthopaedic Research Institute, Bournemouth University, Executive Business Centre, 89 Holdenhurst Road, Bournemouth, BH8 8EB UK; 5 Rigshospitalet, Copenhagen University, Section of Surgical Pathophysiology 7621, Blegdamsvej 9, DK-2100 Copenhagen, Denmark

**Keywords:** Hip arthroplasty, Knee arthroplasty, Rehabilitation, Physical therapy, Exercise therapy

## Abstract

Exercise-based interventions applied before and after total hip and knee arthroplasty (THA and TKA, respectively) have been investigated for a number of years, based on the assumption that they will enhance post-operative recovery. Although recent studies suggest that high-volume, pre-operative exercise may enhance post-operative recovery after TKA, studies of post-operative exercise-based interventions, have not found superiority of one exercise regime over another. It seems, however, that post-operative, exercise-based, rehabilitation is superior to no or minimal rehabilitation after THA and TKA.

The goal of this commentary is to summarize recent evidence for the efficacy of different peri-operative exercise-based interventions to enhance recovery after THA and TKA, and to propose new strategies to further enhance post-operative recovery.

There is a major need to improve functional recovery after THA and TKA. We propose a strategy of “enriched” trials where specific rehabilitation interventions are applied to different patients based on, for example, their expectations for post-operative recovery, willingness to undertake exercise and physical activity, and pre-operative functional performance.

## Background

Total hip and knee arthroplasty (TKA and THA) have been quoted to be some of the most successful operations and performed in an increasing number of patients every year around the world to reduce pain and improve function (Culliford et al. 2015). However, several challenges lie ahead to improve recovery (Aasvang et al. 2015). For example, according to patient-reported outcomes (PROMs), the overall result is generally positive, although discrepancies are seen when PROMs are compared to performance-based function (Luna et al. 2017, Stevens-Lapsley et al. 2011). Also, postoperative activity levels are disappointingly low in many patients, and around 20% of patients are socially isolated following surgery (Harding et al. 2014; Smith et al. 2017). Additionally, about 5 to 20% of all patients report chronic pain after THA and TKA, respectively (Beswick et al. 2012). Given the negative physical and psychological consequences of these factors on outcomes such as all-cause mortality, return to work, and leisure activities, (Smith 2017) there is a significant rehabilitation challenge for this population.

Given these recovery problems, and the fact that there is a large early postoperative loss of knee-extension strength (30 to 80% after THA and TKA, respectively) (Kehlet 2013) major efforts have been paid towards improving post-operative recovery. Since the length of hospital stay and the overall risk of complications have been reduced with the concept of fast-track surgery or enhanced recovery programs, (Jorgensen et al. 2016) the main focus of perioperative care should now be on how to accelerate post-discharge recovery and physical rehabilitation. This work, and the rehabilitation techniques chosen, should also acknowledge that the relative causes and underlying mechanisms for early decreased muscle function are still to be elucidated. Further investigation into the relative role of factors such as oedema, pain, reflex inhibition, and fear, on muscle function in the immediate post-operative phase may help to guide the choice of effective rehabilitation interventions.

Therefore, this commentary will summarize the recent evidence for the efficacy of different peri-operative exercise-based interventions to enhance recovery after THA and TKA, and propose future strategies to further enhance post-operative recovery.

## The current state of research into rehabilitation strategies

With respect to the current evidence for pre and post-operative exercise, we have previously argued that earlier initiated and more intense types of exercise-based physical rehabilitation modalities (e.g. progressive strength training) would likely be superior to less intense (e.g. ADL training without progressive strength training) (Bandholm and Kehlet 2012). However, such superiority has been difficult to demonstrate in both THA and TKA (Bade et al. 2017; Jakobsen et al. 2014; Mikkelsen et al. 2014). Hence, there is currently no firm evidence-base for a single type of exercise-based physical rehabilitation to enhance post-operative recovery in the “average” patient (Artz et al. 2015; Coulter et al. 2013; Haas et al. 2016; Pozzi et al. 2013; Wang et al. 2016).

However, some issues have become clearer in recent years. Firstly, while it has been difficult to demonstrate clinically relevant effects of preoperative exercise on postoperative recovery after THA and TKA (Wang et al. 2016), recent studies using supervised, pre-operative, high-volume, progressive strength training may to some extent enhance the recovery of knee-extension strength and functional performance after TKA (Calatayud et al. 2017; Skoffer et al. 2016). Secondly, exercise-based rehabilitation seems to be superior to no or minimal exercise-based rehabilitation following THA and TKA (Artz et al. 2015; Coulter et al. 2013; Pozzi et al. 2013). Thirdly, home-based physical rehabilitation, i.e. having received one or a few initial exercise instructions, seems equally effective as out-patient, supervised rehabilitation (Artz et al. 2015; Coulter et al. 2013; Pozzi et al. 2013). However, none of these interventions have solved the overall functional recovery problem after these operations. In addition, research to examine specific rehabilitation interventions for patients with known risk factors for slow or impaired functional recovery is particularly lacking.

## Recommendations for future research into rehabilitation strategies

Given the economic resources spent on surgery and subsequent rehabilitation of the “average” patient, (Kjellberg and Kehlet 2016) the question is whether we should change our future cost-effective strategies for improving recovery? In this context – and living in a time with a focus on personalised medicine – we hypothesise that future interventions should be based on a more detailed preoperative characterisation of patients, including: socioeconomic status, expectations regarding recovery, preoperative pain status, psychological status, preoperative fall tendency, and leg muscle-strength status, as these may represent different groups of patients and with potential large differences in post-operative functional recovery. Work to define such groups needs to be undertaken initially in prospective interventional studies.

Consequently, the future strategy may include a specific focus on preoperative exercise (prehabilitation) in patients with reduced preoperative knee-extension strength, impaired functional performance and fall tendency. This will be challenging and so future research to accurately characterise and identify these patients preoperatively is required. Such strategies, in combination with nutritional supplementation for the identified frail/sarcopenic patients may offer enhanced benefits, given the blunted adaptive response of these patients to exercise and nutrition, and the argument that exercise and nutrition are always better together (Phillips 2017). In addition, to exercise and nutrition, educational interventions are also a recognised component of prehabilitation (Carli and Scheede-Bergdahl 2015), and as such should also be investigated, as they may help with changing patient expectations of recovery timescales, and to facilitate patient understanding of how to progress rehabilitation whilst adhering to any surgically imposed restrictions to movements or weight bearing status.

The concept of enriched trials in such “high-risk” patients is different to previous efforts, where trials have been undertaken in “average patients” with limited effect on postoperative recovery in most trials (Bandholm and Kehlet 2012). Such studies should also incorporate methods to capture adherence rates to home exercise, as this may be a critical factor in influencing outcomes (Bollen et al. 2014). Also, preoperative exercise as part of conservative preoperative care for the “average” patient may enable better selection of surgical candidates (Skou et al. 2015).

Returning to exercise-based postoperative physical rehabilitation, it may be preferable to use home-based exercise or community-based exercise classes (which may have additional benefits in regard to social isolation and loneliness) in most patients, following one or more initial exercise instructions (Artz et al. 2015; Coulter et al. 2013; Pozzi et al. 2013). In this context, technology that can objectively monitor exercise adherence and stimulus intensity during home-based exercise is now available, (Rathleff et al. 2014) which enables documentation of exercise adherence and intensity in relation to the degree of observed recovery. Wearable technologies such as activity trackers and smartphone apps also offer much promise within this area, and their value in objectively assessing functional outcomes has begun to be demonstrated (Luna et al. 2017), although further objective evaluation of their specific efficacy when used as a tool to improve outcomes is still required (Bahadori et al. 2018).

Similarly, the promising results of another technology, perioperative neuromuscular electrical stimulation (NMES) requires further investigation (Spector et al. 2016) before recommendations can be provided. The potential role of NMES in accelerating the recovery of muscle function should be evaluated along with its effect using different setting parameters on symptoms such as pain and oedema. It’s use immediately post-surgery (before discharge) may also offer benefits, where by it can enable a high exercise volume, with little effort, at a time point where muscle inhibition is most pronounced. In addition, in post-discharge rehabilitation, its combination with exercise interventions (non-simultaneously) has also been argued as optimal (Vanderthommen and Duchateau 2007).

For all future research of specific rehabilitation interventons, a separation should be made between a “standard” expected good recovering patient and the more difficult patients like those with a continuous subacute or persistent pain problem, those who are on preoperative opioid treatment, (Aasvang et al. 2016) pain catastrophizers, (Lunn et al. 2015) patients receiving preoperative psychopharmacological treatment (Jørgensen et al. 2015) and combined with a detailed assessment of the patient’s preoperative expectations (Haanstra et al. 2012; Nakahara et al. 2015; Palazzo et al. 2014) of their postoperative functional outcome. Such studies will be crucial to understand where we should lay resources for rehabilitation, since many patients have limited daily activity after joint replacement (Harding et al. 2014) and may not be motivated for undertaking greater levels of physical activity – even for expected health gains (Smith et al. 2015). In this case, they may potentially gain little from out-patient physical rehabilitation. Consequently, future strategies should include a focus on needs as well as motivation for physical rehabilitation. There may be patients who will recover by themselves by actively engaging in simple, inexpensive, home-based rehabilitation; patients who may not want to actively engage in any type of physical rehabilitation and have no major expectations for functional recovery besides pain alleviation; and “high-risk” patients who need much more intensive and supervised physical rehabilitation.

Given the recommendations provided, an important future step may also be the transfer of recent research evidence into practical clinical guidelines, that can help to guide clinicians and support the implementation of evidence to everyday clinical practice. Such guidance does not currently exist for physical rehabilitation after THA and TKA and may be a useful adjunct to increase the impact of future clinical trials.

## Conclusions

In summary, there is a continuous major need to improve functional recovery after hip and knee replacement, because studies to date have not found superiority of one exercise regime over another. However, whilst exercise-based rehabilitation seems superior to no or minimal rehabilitation after THA and TKA, future rehabilitation strategies will require us to examine different patient groups and the use of specific postoperative rehabilitation interventions based within “enriched” trial designs.

## Abbreviations

NMES: Neuromuscular electrical stimulation
PROMs: Patient reported outcome measures
THA: Total hip arthroplasty
TKA: Total knee arthroplasty
